# Bismuth Film along with dsDNA-Modified Electrode Surfaces as Promising (bio)Sensors in the Analysis of Heavy Metals in Soils

**DOI:** 10.3390/bios14060310

**Published:** 2024-06-18

**Authors:** Vasiliki Keramari, Sotiria G. Papadimou, Evangelia E. Golia, Stella Girousi

**Affiliations:** 1 Analytical Chemistry Laboratory, School of Chemistry, Aristotle University of Thessaloniki, 541 24 Thessaloniki, Greece; 2 Laboratory of Soil Science, School of Agriculture, Aristotle University of Thessaloniki, 541 24 Thessaloniki, Greece; sotiriapg@auth.gr (S.G.P.); egolia@auth.gr (E.E.G.)

**Keywords:** heavy metals, bismuth film electrode, dsDNA-modified electrode, anodic stripping voltammetry, atomic absorption spectroscopy, soil samples

## Abstract

Heavy metals constitute pollutants that are particularly common in air, water, and soil. They are present in both urban and rural environments, on land, and in marine ecosystems, where they cause serious environmental problems since they do not degrade easily, remain almost unchanged for long periods, and bioaccumulate. The detection and especially the quantification of metals require a systematic process. Regular monitoring is necessary because of seasonal variations in metal levels. Consequently, there is a significant need for rapid and low-cost metal determination methods. In this study, we compare and analytically validate absorption spectrometry with a sensitive voltammetric method, which uses a bismuth film-plated electrode surface and applies stripping voltammetry. Atomic absorption spectroscopy (AAS) represents a well-established analytical technique, while the applicability of anodic stripping voltammetry (ASV) in complicated sample matrices such as soil samples is currently unknown. This sample-handling challenge is investigated in the present study. The results show that the AAS and ASV methods were satisfactorily correlated and showed that the metal concentration in soils was lower than the limit values but with an increasing trend. Therefore, continuous monitoring of metal levels in the urban complex of a city is necessary and a matter of great importance. The limits of detection of cadmium (Cd) were lower when using the stripping voltammetry (SWASV) graphite furnace technique compared with those obtained with AAS when using the graphite furnace. However, when using flame atomic absorption spectroscopy (flame-AAS), the measurements tended to overestimate the concentration of Cd compared with the values found using SWASV. This highlights the differences in sensitivity and accuracy between these analytical methods for detecting Cd. The SWASV method has the advantage of being cheaper and faster, enabling the simultaneous determination of heavy elements across the range of concentrations that these elements can occur in Mediterranean soils. Additionally, a dsDNA biosensor is suggested for the discrimination of Cu(I) along with Cu(II) based on the oxidation peak of guanine, and adenine residues can be applied in the redox speciation analysis of copper in soil, which represents an issue of great importance.

## 1. Introduction

Heavy metals constitute an environmental health risk that humanity has been concerned about for the last decades [1,2]. Several of them in small quantities (trace amounts), such as copper (Cu) and zinc (Zn), are essential for the smooth and safe growth and reproduction processes of living beings [3]. However, once they reach hazardous concentrations, they pose a risk both to the environment and plant or animal organisms they are present in [4]. Meanwhile, a few metals such as cadmium (Cd) and lead (Pb) have no beneficial effect on living organisms, even when present in small quantities. Probably the greatest problem is that heavy metals have the potential to remain unchanged for long periods, as they have long half-lives [5]. They also bioaccumulate in both the abiotic environment and in living organisms, causing numerous diseases, such as neurological disorders, cancer, and even death [6]. Their presence is widespread on land and in the sea, as well as in the air, water, and soil. It is well known that soils are subject to long-term pollution, and, therefore, the ongoing recording and monitoring of their concentrations is necessary [7]. The sources of their origin are numerous, with the following two major classes dominating: geochemical and anthropogenic. Minerals and rocks in soils constitute the main source of their origin, as they are decomposed by physical (air, rain, ice), chemical (oxidation, hydrolysis), and other factors. However, human activities, industrial processes, heating fuel use, exhaust gases from wheeled vehicles, etc., generate heavy metal pollution in the mainly urban environment [8,9]. In agricultural soils, the indiscriminate use of fertilizers and plant protection products along with biological treatment sludge often contain heavy metals in small or large quantities.

Soil contains multiple heavy metals, such as Zn^2+^, Cu^2+^, Pb^2+^, Cd^2+^, A^g+^, and As^3+^/As^5+^. The concentrations of Cu^2+^ and Zn^2+^ in the soil are remarkably higher than those of Pb^2+^ and Cd^2+^ [10,11], which is due to complex and alloy formation [12].

Over time, a range of techniques has been developed for detecting heavy metal ions (HMIs) in soil samples, each with its own set of advantages and limitations, including inductively coupled plasma mass spectrometry (ICP-MS) [12,13], inductively coupled plasma optical emission spectrometry (ICP-OES) [14], atomic absorption spectroscopy (AAS) [15,16], and atomic absorption spectroscopy (AAS) [17,18,19]. These methods vary in their focus, sometimes emphasizing parameters and other times the choice of analysis method. While spectrometric methods like AAS and ICP-MS offer exceptional precision and responsiveness, they come with drawbacks such as high cost, time-dependent behavior, and the inability to conduct on-site measurements. Thus, selecting the appropriate technique depends on factors like required precision, sensitivity responsiveness, cost considerations, and the feasibility of on-site analysis.

Although these techniques offer high sensitivity and selectivity, they have several notable limitations. On the other hand, electrochemical techniques like voltammetry offer distinct advantages including lower cost, ease of use, rapid analysis, simple portability, and suitability for field monitoring of environmental samples, as well as high sensitivity and selectivity [20,21].

Voltammetry stands out among other electrochemical techniques as the leading electrochemical method for its exceptional sensitivity and selectivity, making it capable of in situ simultaneous determination and measurement of heavy metals in different soil samples [20]. Cyclic voltammetry (CV) and electrochemical impedance spectroscopy are mainly applied to electrode surface characterization [22], while square wave voltammetry (SWV) [23], linear scan voltammetry (LSV), and differential pulse voltammetry (DPV) are applied in the determination [24]. Different pulse techniques are discriminated by the method of the applied potential (linear sweep, square wave, or differential pulse) [25]. It has been reported that the simultaneous electrochemical determination of Cd^2+^, Pb^2+^, Cu^2+^, and Zn^2+^ in soil samples, with a glassy carbon electrode (GCE) modified with a bismuth, film can be achieved using differential anodic stripping pulse voltammetry (DP-ASV) [26].

Additionally, DPV and SWV exhibit better detection responsiveness than the other techniques. In addition, electroanalysis offers promising analytical methodologies for the determination of several metal ions. It is worth mentioning that electroanalysis can also be easily combined with inexpensive and user-friendly instruments.

The square wave anodic stripping voltammetry (SWASV) electroanalytical technique for the detection of Zn^2+^, Cd^2+^, Cu^2+^, and Pb^2+^, is considered a promising analytical tool because of its improved analytical characteristics in the determination of heavy metals of considerable concern. On the other hand, certain ions interfere with the determination of heavy metals [27]. An electrode can be modified for a specific purpose of use.

During the last years, chemically modified electrodes have offered remarkable and advantageous prospects in chemical analysis. Chemical modification involves carbon materials along with bio-elements such as enzymes, deoxyribonucleic acid, etc.

Because of its characteristics, in this experiment, bismuth is chosen as a film on electrodes, such as glassy carbon electrodes (GCEs), which find applications in various sample analyses (environmental, biological, etc.). Carbon electrodes, mainly glassy carbon and carbon paste, offer an important platform for the development of modified electrode surfaces based on bismuth film, which restores the merits of mercury film excluding its toxicity. The unique character of bismuth film electrodes has led to the growing importance of its application in chemical analysis [28].

The purpose of this paper is to present a simple and easy-to-apply analytical method for the preparation of a glassy carbon electrode modified with bismuth film that can be used for the sensitive and selective simultaneous voltammetric determination of the total concentration of cadmium, lead, zinc, and copper in soil samples using square wave voltammetry. Moreover, in this work, an electrochemical biosensor is suggested to differentiate the behavior of Cu(I) and Cu(II) with double-stranded DNA, which results in a sensitive and selective analytical tool for redox copper speciation in soil.

The novelty of this paper is based on the following facts: The analytical behavior of a bismuth-film electrode (BFE) was investigated, for the first time, for the simultaneous determination of four metal ions, including Zn(II), Pb(II), Cd(II), and Cu(II), in soils of both rural and urban origin. The proposed analytical methodology is specific, sensitive, and reproducible. The analytical figures of merit are shown in Table 3.

## 2. Materials and Methods

### 2.1. Handling of Samples

The soil samples included in this study were divided into three different groups as follows: natural soils derived from agricultural soils that were intensively cultivated for years [7], soils from the urban fabric of crowded cities [29,30], and soils that were artificially or laboratory-contaminated [31] to achieve high concentrations of metals, particularly cadmium, that were considerably higher than usual in soils [1,2]. The soil samples from rural and urban areas were contaminated (spiked) by adding appropriate volumes of metal nitrate solutions. The solutions were mixed with the soil samples by spraying and mechanical agitation for 15 min. They were then transferred to black plastic bags and kept there to incubate for a month. Aeration was applied to maintain the moisture content at the 50% level.

Both the naturally contaminated and spiked soils were transferred to the Laboratory of Soil Science at the Aristotle University of Thessaloniki, where they were subjected to soil analyses after being air-dried and passed through a 2mm diameter sieve. The methodology described by [7,32] was then followed.

Nutrient concentration levels were measured according to the related literature [32,33]. Soil texture and soil–water mixture measurements in a ratio of 1:2.5 were used to determine the pH value according to Vougioukos’ method [33]. For the electrical conductivity (EC) measurements, a soil–water saturation paste was used at an equal ratio, i.e., 1:1. Total nitrogen was measured by the Kjehldal method, and assimilable phosphorus by the Olsen method. The concentration of exchangeable potassium was evaluated by applying a Flame Photometer (Sherwood 410) after extraction with 0.1 M ammonium acetate, pH = 7. The organic matter content of the soil samples as well as their mixtures was obtained by the Walkley–Black method [34].

Total metal concentrations were determined after extraction using Aqua Regia (HCl:HNO_3_, 3:1) [35]. An atomic absorption spectrometer equipped with a flame (Shimatzu 6300) or graphite furnace (Shimatzu 7000) was used. Certified reference material CRM 141R was analyzed. The certified concentrations for Cu, Zn, Cd, and Pb were 0.29, 0.68, 0.019, and 0.218 μg·L^−1^, respectively, while the precision for metal analysis ranged from 7.8 to 10.9%.

For the voltammetric measurements, the digested samples were further diluted with deionized water (1:5). Voltammetric measurements were realized using a Palm Sens Model 1 potentiostat/galvanostat from Echo Chemie in the Netherlands, where heavy metal determinations were performed. The electrochemical cell used in this experiment included a glassy carbon working electrode (GCE), an Ag/AgCl reference electrode (RE) saturated with 3 mol·L^−1^ KCl, and a platinum wire counter electrode (CE). The electrodes were used to determine heavy metals, more specifically, cadmium (Cd), lead (Pb), copper (Cu), and zinc (Zn). A Sartorius balance and a Consort C830 pH meter were used for relevant measurements. A magnetic stirring bar 8 × 3 mm, PTFE (HEINZ HERENZ HAMBURG) was used.

Solvents, acids, bases, and standard solutions were all pro-analysis grade. The supporting electrolyte in anodic stripping voltammetry (ASV) was 0.1 mol L^−1^ acetate buffer (pH = 4.5), prepared from acetic acid, and sodium hydroxide (CH_3_COOH/CH_3_COONa) (ACS reagent, Darmstadt, Germany) provided by Merck. Standard solutions of bismuth, cadmium Cd, lead Pb, copper Cu, and zinc Zn were provided by Sigma-Aldrich (Darmstadt, Germany). Hydrogen peroxide (30 volume%) was pro-analysis grade from Μerck. Double-stranded (ds) calf thymus DNA (D-1501, highly polymerized) was purchased from Sigma. To study the electrochemical behavior of dsDNA at the electrode surface, stock solutions (0.14 g·L^−1^) were prepared in 10 mM Tris–HCl and 1 mM EDTA at pH 8.0. All aqueous solutions were prepared using deionized water. The experiment aimed to determine heavy metals such as Cd, Pb, Cu, and Zn in soil samples by using Bi(III) as the substrate of the GCE electrode. The soil solutions were diluted 1:5 in 25 mL volumetric flasks.

The voltammetric procedure aimed to determine cadmium, lead, copper, and zinc using standard solutions with known concentrations of these metals. The technique involves square wave voltammetry and in situ plating. The process consists of the following steps:

1. Plating: The electrode surface is prepared by applying an acetate buffer, Bi(III), and H_2_O_2_. The electrode is then plated at a deposition potential of −0.3 V for 60 s. This step creates a bismuth film on the electrode surface, which enhances the sensitivity of the measurements.
2. Preconcentration: Metal ions (cadmium, lead, copper, and zinc) are preconcentrated onto the plated electrode surface with a deposition potential of −1.4 V for 60 s. This step accumulates the metal ions on the electrode, followed by an equilibration period of 10 s to stabilize the system before measurement.
3. Conditioning: After preconcentration, the electrode is conditioned by applying a potential of 0.3 V for 30 s. This step prepares the electrode for square wave voltammetry (SWV) experiments, ensuring that the surface is in an optimal state for the accurate detection of metal ions.

First, the glassy carbon electrode was rinsed with deionized water, and then its surface was polished with 0.1 mm and 0.05 mm alumina powder since a polishing cloth was required to produce a mirror-like surface. A 0.1 mol·L^−1^ acetate buffer (CH_3_COOH/CH_3_COONa), pH 4.7, was used as a supporting electrolyte in square wave voltammetry (SWV). The electrode surface was modified by a bismuth film electrode. The potential was recorded in the range from −1.400 to +0.0 V, with a potential step of 5 mV, a pulse potential of 15 mV, and a frequency of 10 Hz. Magnetic stirring was used as needed.

### 2.2. dsDNA-Modified Electrode

The dsDNA-modified electrode preparation was realized by applying pre-treatment at +1.7 V for 1 min at the bare electrode surface. In comparison, dsDNA was subsequently immobilized at the electrode surface for 5 min at +0.5 V. Measurements were recorded in 0.2 M acetate buffer solution scanning from +0.1 V to the positive potential value direction in differential pulse voltammetry mode (DPV) with a scan rate of 50 mV·s^−1^. Details are given elsewhere [36,37].

The results were statistically processed by applying the SPSS package (IBM SPSS Statistics 26) and Microsoft Office Excel. Every experiment was performed five times. Fisher’s least significant difference (LSD) test at *p* = 0.05 was performed.

## 3. Results and Discussion

### 3.1. Physico-Chemical Features of Soil Samples

Table 1 presents the physico-chemical properties of the soil samples.

Both skewness and kurtosis are statistical variables with a variety of applications, but mostly they are used to describe the characteristics of a distribution. Skewness is a tool for expressing either positive or negative symmetry in a dataset. In particular, when the value is −0.5 or 0.5, the value distribution is almost symmetrical. The data are positively skewed if the value is between 0.5 and 1 and negatively skewed if it is between −1 and −0.5. On the other hand, kurtosis accounts for the tail of a distribution, as it quantifies the outliers in the distribution under investigation. In comparison with a normal distribution, the dataset displays lighter tails if kurtosis is less than 3 [38].

The soil samples chosen for this study are alkaline. Furthermore, it is well known that in urban and peri-urban areas, soils tend to become alkaline over time [30,39]. The electrical conductivity and organic matter values suggest common values found in agricultural and urban soils investigated in [40]. The highest electrical conductivity values were observed in the following cases: in urban soil samples located around city ports and in agricultural soils derived from samples that were exposed to high amounts of fertilizers [7,29]. Soils also exhibit great variability concerning the values of clay and sand. Mónok et al. [8] studied and compared the physico-chemical properties of urban soils with cultivated soils and concluded that the properties of urban soils show considerable similarities among them, often following a known and recurring pattern.

In Table 2, the nutrients along with the toxic metal concentrations are presented.

Lu et al. [39] investigated the consequences of pH fluctuations, mainly caused by endogenous redox reactions and soil buffering capacity, on Cd(II) speciation in rice paddy soils when flooding and drying cycles occur. Lue et al. [42] also investigated the potential factors, such as soil pH, and the extent of urban green space and climatic conditions, that may determine and ultimately mitigate the accumulation of toxic heavy metals in urban soils. Skorbiłowicz et al. [43] studied the concentrations of potentially toxic elements observed in soils around the perimeter of high-traffic roads on the Bialystok–Budzisko route in Northeastern Poland, where significantly high concentrations of Pb and Cd are present. In their study, the naturally contaminated soil samples were found to have concentrations lower than the maximum permissible limits established by the European Union [41]. The metal values were much higher in the spiked soils, as these concentrations are hardly found in real life, even in mines, and would be a significant risk to people’s health [30,44,45]. However, the presented concentrations were selected to meet the requirements of investigating the limitations of the quantitative determination of metals using certain analytical techniques.

### 3.2. Voltammetric Determination

#### 3.2.1. Bismuth Film-Modified Electrode

Since the year 2000, bismuth film-modified electrodes have been attracting increasing attention in electroanalysis. In this context, bismuth film electrodes (Bi-FEs) have become an attractive new subject of electro-analytical research as a potential replacement for mercury electrodes. Bismuth is known as a “green metal” and represents a competitive alternative to mercury, which is known to be highly toxic. Sensors based on bismuth film modification procedures offer a remarkable enhancement in responsiveness, selectivity, measurement flexibility, as well as analytical figures of merit, enabling them to become very competitive.

Bi-film electrodes (BFEs) are prepared by depositing a thin layer of bismuth onto a suitable substrate material instead of using mercury. The primary advantage of BFEs lies in their eco-friendliness, as bismuth and its salts are minimally toxic. Furthermore, BFEs exhibit analytical characteristics in voltammetric analysis that are roughly equivalent to those of mercury film electrodes (MFEs). This similarity is attributed to bismuth’s ability to form “fused alloys” with heavy metals, akin to the amalgams formed by mercury [46].

The electrode modification process with bismuth can be accomplished through two distinct methods as follows:

- Plating bismuth film in a supporting electrolyte solution different from that of measurement (ex situ).
- Plating bismuth film in the same supporting electrolyte solution as that of measurement (in situ) [47].

In Table 3, the already established analytical methodologies are summarized and compared. The proposed analytical methodology is unique as it allows for the simultaneous determination of Zn(II), Pb(II), Cd(II), and Cu(II) in soils.

Their primary limitation lies in their restricted anodic range, which hinders their utility in detecting or accumulating species at higher positive potentials. Overall, it can be concluded that BFEs represent a useful analytical tool and indeed have the potential to replace Hg-film electrodes (MFEs) [46].

Extensive research indicates that the detection of heavy metals using BFE electrodes relies on bismuth’s ability to form a “fused alloy” with metals like lead, cadmium, and zinc [46]. The electrode surface is bismuth film plated in situ, which is parallel to the determined metal ions during the preconcentration step in anodic stripping voltammetry, from a solution containing added Bi(III) ions, or ex situ, where bismuth film can be formed in an external plating solution [47].

For the determination of heavy metals (Zn, Cd, Pb, Cu), we used square wave anodic stripping voltammetry at a bismuth film-modified glassy carbon electrode. The process of accumulation (electrolysis) is realized across the electrochemical reduction of a marked substance by invariable potential, joint from a simultaneous mixing solution. Thanks to high sensitivity and low limits of detection, this method finds use in the analysis of many natural samples as well as industrial samples.

The electrode surface is bismuth film plated in parallel with the determined metal ions during the preconcentration step in anodic stripping voltammetry, from a solution containing added Bi(III) ions, or ex situ, where bismuth film can be formed in an external plating solution.

Next, we completed measurements following potentiostatic accumulation and the equilibrium period.

During ex situ plating, increasing copper concentration was followed by a significant decrease in the bismuth signal. Nearby the electrode surface, competition for surface sites took place between the deposited copper and bismuth following a significant overlap in bismuth and copper signals.

After bismuth in situ plating, the electrode surface responsiveness was remarkably improved, giving rise to higher Cd, Pb, and Zn peaks, while between bismuth and copper, the separation was not favored since they competed for the available electrode surface sites. In our previous work [62,63], which involved the simultaneous determination of Zn, Cd, Pb, and Cu using BFE in samples of biological and environmental concern, the problem of the simultaneous determination of Cu along with Zn, Cd, and Pb was studied either by the addition of Ga(III) or by the addition of H_2_O_2_. In the case of the addition of Ga(III), the linear range for copper was more limited than the corresponding range obtained after the addition of H_2_O_2_.

The addition of H_2_O_2_ favored the simultaneous determination because of the following reasons:

(a) It causes a noticeable shift in the redissolution peak of copper to more positive potential values (at +0.212 V) than expected (at −0.100 V) [redissolution improvement].
(b) It eliminates competition between Bi(III) and Cu(II) for free electrode surface sites (interference minimization).
(c) It ensures an excellent improved correlation between the copper concentration and its peak current (r > 0.99).
(d) It achieves lower limits of detection Cu(II).
(e) It gives satisfactory repeatability for consecutive measurements (n = 10).
(f) It guarantees excellent recovery performance (96–108%).

For the reasons given above, the in situ modification of the GCE electrode surface was preferred over the ex situ process for the simultaneous determination of Cd, Pb, Cu, and Zn because it gives more reliable results.

**Figure 1:**
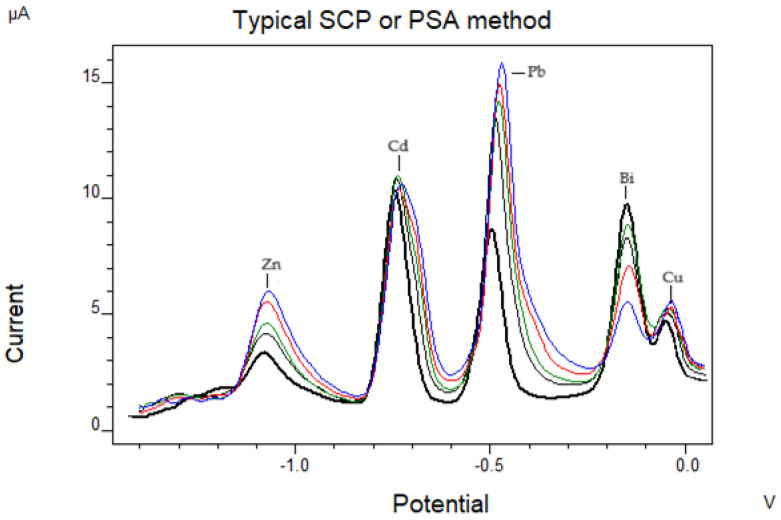
Voltammogram showing the simultaneous determination of Cu, Zn, Pb, and Cd: 50 μL, 250 μL, −1.4 V for 120 s, scanning from −1.4 V (E_begin_) to +0.0 V (Eend), 0.12% *v*/*v* H_2_O_2_, 100 μg/L Bi(III), buffer CH_3_COOH/CH_3_COONa (Ph = 4.7), 10 Hz (SW frequency), 0.005 V (Estep), 0.015 V (Epulse). (Black line: 1st sample addition, gray line: 2nd sample addition, green line: 3rd sample addition, red line: 4th sample addition, blue line: 5th sample addition).

Applying the procedure of in situ plating, the limits of detection were 0.70 mg/L for Zn, 0.14 mg/L for Cd, 0.03 mg/L for Pb, and 0.38 mg/L for Cu (measured values in the digest), and the relative standard deviations were 8.60% for Zn, 4.75% for Cd, 5.68% for Pb, and 5.38% for Cu at the 2.0 mg/L level (n = 5). A voltammogram of the simultaneous determination is shown in Figure 1.

The method’s efficiency was verified using CRM 141R standard soil. For Zn, Cd, Cu, and Pb, the method quantification limit (MQL) values were 0.91, 0.88, 1.1, and 0.88 mg kg^−1^, respectively, while the precision for metal analysis ranged from 8.2 to 10.4%.

**Figure 2:**
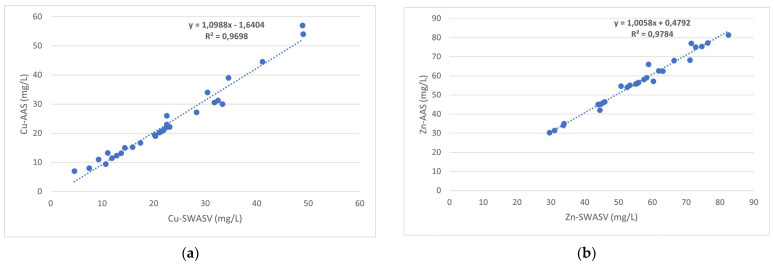

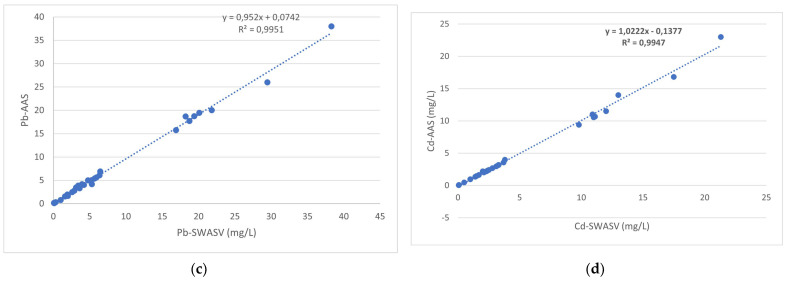
Comparison of AAS and SWASV with the bismuth film-modified electrode: (**a**) Cu; (**b**) Zn; (**c**) Pb; and (**d**) Cd.

Moreover, the analytical method was validated by applying atomic absorption spectrometry. The results shown in Figure 2 proved to be in good agreement (ANOVA, Student’s test, 95% confidence level). This indicates that the method is unbiased and proves its validity and versatility.

#### 3.2.2. dsDNA-Modified Electrode

According to our previous work [36,37], it is possible to perform the redox speciation of copper based on the interaction between dsDNA and Cu(I) and Cu(II).

The interaction between Cu(II) and dsDNA resulted in an increase in the characteristic oxidation peak of guanine and a decrease in the adenine oxidation peak. The interaction phenomenon proved to be time-dependent.

**Figure 3:**
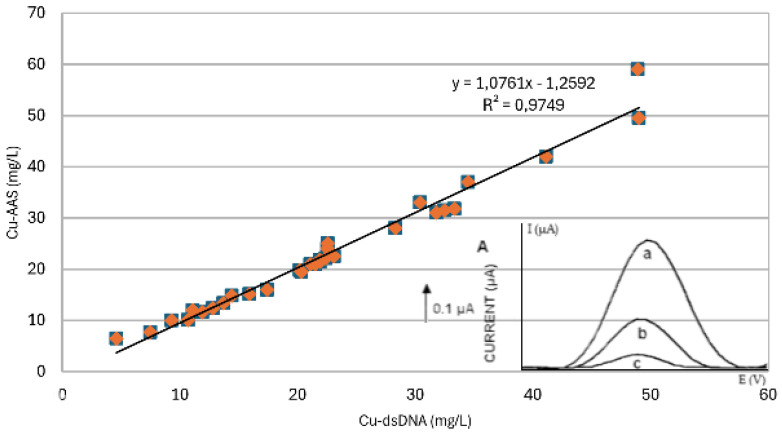
Comparison of AAS and SWASV with the dsDNA-modified electrode (based on guanine oxidation peak) regarding Cu(II). Inset: voltammogram of guanine addition.

In the case of Cu(I), dsDNA was electrostatically adsorbed at the electrode surface, while their interaction resulted in a peak at 1.37 V, which started to appear with increasing concentrations of Cu(I). The proposed (bio)sensor can be used for copper speciation in soil samples. Figure 3 shows a comparison of AAS and dsDNA-modified electrodes (based on guanine oxidation peak) regarding Cu(II). 

The limit of detection for Cu(II) equalled 0.019 mg/L (measured values in the digest), with a relative standard deviation of 6.67% at the 2.0 mg/L level (n = 5).

In CRM 141R standard soil, by applying the dsDNA-modified electrode, the Cu(II) concentration was found to be 0.97 mg kg^−1^ with an RSD value of 9.9%. Regarding Cu(II)/Cu(I) redox speciation in the same certified standard soil CRM 141R (spiked with Cu(I)), a recovery of 91.7% Cu(I) was obtained with an RSD value of 8.7%.

## 4. Conclusions

Heavy metals are prevalent pollutants in air, water, and soil that are found in both urban and rural environments, as well as terrestrial and marine ecosystems. Their persistence and bioaccumulation, due to their resistance to degradation, pose significant environmental challenges. Their detection and quantification is a systematic process, as monitoring their levels needs to be carried out at regular intervals since there is often seasonal variation. The need for rapid and low-cost determination of total metal concentration as well as speciation of copper is therefore considerable. In the present work, the quantitative determination of Cu, Cd, Pb, and Zn was carried out in extracts of soil samples obtained from the urban area of Thessaloniki. Metals were extracted using an Aqua Regia mixture and subsequently determined using two analytical techniques as follows: atomic absorption spectroscopy (AAS), using a flame or graphite furnace fixture, and square wave anodic stripping voltammetry (SWASV), using a bismuth thin film electrode.

Regarding the quantification of Cu, Cd, Pb, and Zn, the values determined by both methods are in satisfactory agreement. Regarding Cd, it was apparent that the limits of detection of Cd were lower with AAS than with SWASV when using the graphite furnace, while an overestimation was noted when using flame-AAS in comparison with those found with SWASV. The SWASV method is advantageous as it is cheaper and faster, enabling the simultaneous determination of all four elements across the range of concentrations that these elements can occur in Mediterranean soils. Additionally, a dsDNA-modified electrode surface is proposed as a promising analytical tool/biosensor for copper speciation.

More specifically, the proposed analytical methodology was validated using a certified reference material, i.e., CRM 141R standard soil. Based on the results, the method quantification limit (MQL) values for Zn, Cd, Cu, and Pb were 0.91, 0.88, 1.1, and 0.88 mg kg^−1^, respectively, while the precision for metal analysis ranged from 8.2 to 10.4%. Additionally, regarding the dsDNA-modified electrode, the Cu(II) concentration was found to be 0.97 mg kg^−1^ with an RSD value of 9.9%. Regarding Cu(II)/Cu(I) redox speciation in the same certified standard soil, i.e., CRM 141R (spiked with Cu(I)), a recovery of 91.7% Cu(I) was obtained with an RSD value of 8.7%.

## Figures and Tables

**Table 1:** Values of the physico-chemical parameters of the soil samples (n = 30).

	pH (1:1)	EC (μS cm^−1^)	OM (%)	Clay (%)	Sand (%)	CaCO_3_ (%)
Minimum value	7.12	1224	0.55	11	21	8.65
Maximum value	8.82	4431	3.43	56	67	19.34
Mean value	7.49	2205	1.97	24	44	11.33
Relative standard deviation	0.55	11.13	0.88	2.30	6.43	1.33
Kurtosis coefficient	1.339	−0.385	−0.543	−0.008	−0.082	−0.760
Skewness coefficient	0.690	0.643	−0.213	1.0088	−0.944	0.331

**Table 2:** Values of nutrients and heavy metal concentrations in the soil samples (n = 30).

	N	P	K	Cu	Zn	Pb	Cd
	(%)	(mg·kg^−1^)	(mg·kg^−1^)	(mg·kg^−1^)	(mg·kg^−1^)	(mg·kg^−1^)	(mg·kg^−1^)
Minimum value	0.1	7.81	45.12	17.44	28.13	4.54	0.17
10th perc ^a^	0.12	10.41	57.61	19.45	33.14	9.15	0.24
50th perc ^b^	0.15	14.66	66.87	26.78	47.31	28.55	0.76
Average	0.19	16.62	71.77	29.88	49.02	29.93	0.88
90th perc ^c^	0.2	17.67	88.89	32.45	52.07	39.72	0.91
Maximum value	0.22	22.77	97.73	34.76	69.11	43.54	1.05
EU Limits ^d^				140	300	300	3

^a^ 10th percentile; ^b^ 50th percentile; ^c^ 90th percentile; ^d^ 86/278/EEC Directive [41].

**Table 3:** Comparison of the proposed methodology with already established analytical methodologies.

		Detection Limit (μg·L^−1^)	Metals for Detection			
		R.S.D.s (%)	Cd	Pb	Zn	Cu
**This work**	SWASV	LOD	0.14	0.03	0.70	0.38
		s_r_%	4.75	5.68	8.60	5.38
**References**	[48]	LOD	-	0.3	-	-
		s_r_%	-	7.4	-	-
	[49]	LOD	-	-	-	5.00
		s_r_%	-	-	-	2.00
	[50]	LOD	0.30	0.40	0.40	-
		s_r_%	3.00	3.20	2.60	
	[51]	LOD	3.20	-	-	-
		s_r_%	3.90	-	-	-
	[52]	LOD	1.20	0.90	-	-
		s_r_%	5.60	6.00	-	-
	[53]	LOD	0.69	0.89	54.00	-
		s_r_%	5.40	6.30	8.80	-
	[54]	LOD	1.00	0.5	-	-
		s_r_%	3.70	4.40	-	-
	[55]	LOD	9.30	8.00	-	-
		s_r_%	2.00	7.00	-	-
	[56]	LOD	3.60	2.50	8.20	-
		s_r_%	2.20	1.50	4.90	-
	[57]	LOD	0.45	0.41	0.52	-
		s_r_%	7.50	10.50	6.00	-
	[58]	LOD	0.27	0.11	-	-
		s_r_%	8.70	3.67	-	-
	[59]	LOD	0.57	0.38	-	-
		s_r_%	9.00	4.50	-	-
	[60]	LOD	7.00	5.00	-	-
		s_r_%	10.14	11.18	-	-
	[61]	LOD	0.003	0.037	0.05	-
		s_r_%	-	-	-	-

## Data Availability

The data that support the findings of this study are available from the corresponding author upon reasonable request.
